# Targeting non-coding RNAs in the ferroptosis system: Molecular mechanisms and clinical translation for reversing doxorubicin resistance in breast cancer

**DOI:** 10.1016/j.ncrna.2026.04.003

**Published:** 2026-05-12

**Authors:** Xiaojie Yu, An Xu, Longdi Yao, Xiao Huang, Jianwen Wang, Deyuan Fu

**Affiliations:** a Department of General Surgery, Northern Jiangsu People's Hospital Affiliated to Yangzhou University, Yangzhou, Jiangsu, 225000, PR China; b Department of Thyroid and Breast Surgery, Changxing Hospital of Traditional Chinese Medicine, Huzhou, Zhejiang, 313000, PR China; c Department of Thyroid and Breast Surgery, Xinghua People's Hospital Affiliated to Yangzhou University, Xinghua, Jiangsu, 225700, PR China

**Keywords:** Breast cancer, Chemoresistance, Doxorubicin, Ferroptosis, Non-coding RNAs

## Abstract

Breast cancer remains one of the leading causes of cancer-related morbidity and mortality in women worldwide, and the clinical efficacy of doxorubicin (DOX) is frequently compromised by chemoresistance. Ferroptosis, an iron-dependent form of regulated cell death characterized by excessive lipid peroxidation, has recently emerged as a critical determinant of tumor therapeutic response. At the same time, non-coding RNAs (ncRNAs), including microRNAs (miRNAs), long non-coding RNAs (lncRNAs), and circular RNAs (circRNAs), have been increasingly recognized as important regulators of gene expression, tumor progression, and drug sensitivity. This review summarizes current advances in understanding how ncRNAs modulate DOX resistance in breast cancer through ferroptosis-related pathways. We first outline the core mechanisms of ferroptosis, including dysregulated iron metabolism, lipid peroxide accumulation, and impaired antioxidant defenses, together with the major molecular bases of DOX resistance. We then highlight the distinct yet interconnected roles of miRNAs, lncRNAs, and circRNAs in regulating ferroptotic vulnerability. Emerging evidence indicates that aberrant ncRNA expression promotes a ferroptosis-resistant phenotype by strengthening the System Xc^−^–GSH–GPX4 antioxidant axis, activating the FSP1–CoQ10 pathway, and reprogramming iron homeostasis, thereby contributing to DOX resistance. Conversely, targeting specific ncRNAs can restore ferroptotic sensitivity and resensitize breast cancer cells to DOX. From a translational perspective, ncRNAs hold promise both as minimally invasive biomarkers for predicting therapeutic response and monitoring resistance, and as therapeutic targets for RNA-based or nanotechnology-enabled combination strategies. Nevertheless, major challenges remain, including compensatory ferroptosis networks, molecular subtype heterogeneity, limited delivery efficiency, and biosafety concerns. Overall, the ncRNA–ferroptosis axis represents a promising mechanistic framework and translational avenue for overcoming DOX resistance in breast cancer.

## Graphical abstract

**Figure:**
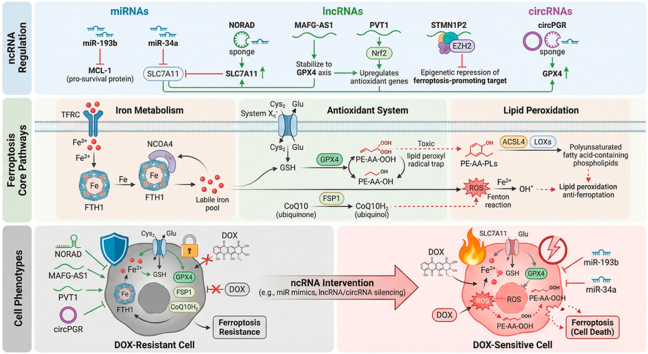

## 1 Introduction

Breast cancer remains one of the most prevalent and life-threatening malignancies for women worldwide, with a growing global burden. In 2023, there were over 2.3 million new cases diagnosed globally, 18% of which occurred in China [1], reflecting persistently high incidence and mortality that underscore breast cancer as a major public health challenge. In systemic breast cancer treatment, chemotherapy remains a cornerstone of comprehensive management. Among chemotherapeutic agents, the anthracycline doxorubicin (DOX) plays a central role in standard regimens, primarily through its core mechanism of DNA intercalation and topoisomerase II inhibition, leading to apoptosis. DOX is indispensable in both neoadjuvant therapy and advanced disease settings [2]. However, its clinical utility is substantially constrained by the development of drug resistance. Epidemiological data indicate that primary DOX resistance occurs in 35%–50% of breast cancer cases overall, rising to 50%–60% in refractory subtypes such as triple-negative breast cancer [3], establishing resistance as a key contributor to treatment failure and poor prognosis. Conventional explanations for DOX resistance include drug efflux pump overexpression (e.g., P-glycoprotein), enhanced DNA damage repair, and apoptosis evasion [[4], [5], [6]]. Despite extensive research targeting these pathways, clinically reversing resistance remains challenging, highlighting the urgent need to identify novel mechanisms and develop innovative therapeutic approaches.

In recent years, the convergence of two rapidly evolving research fields has opened new avenues for overcoming chemoresistance. The conceptualization of ferroptosis in 2012 marked a turning point in resistance mechanisms research. As an iron-dependent, lipid peroxidation-driven form of regulated cell death, ferroptosis is fundamentally distinct from apoptosis, necrosis, and autophagy in both biochemical properties and morphological features [7,8]. Accumulating evidence has established ferroptosis as a critical mediator of the antitumor effects of various chemotherapeutic agents, including doxorubicin [9]. Drug-resistant tumor cells frequently evade cytotoxicity by reinforcing their ferroptosis defense mechanisms, primarily through the System Xc^−^–GSH–GPX4 antioxidant axis, the FSP1-CoQ10 compensatory pathway, and intracellular iron homeostasis regulation. Notably, multiple studies have demonstrated that targeted induction of ferroptosis can effectively eliminate chemotherapy-resistant tumor cells [10], highlighting its substantial therapeutic potential for overcoming drug resistance.

Concurrently, the emerging understanding of non-coding RNAs (ncRNAs) has provided critical insights into the relationship between ferroptosis defense mechanisms and drug resistance [11]. Initially considered "transcriptional noise," ncRNAs are now established as essential regulators of gene expression at epigenetic, transcriptional, and post-transcriptional levels. Beyond their general roles in gene regulation, ncRNAs are increasingly recognized as important modulators of regulated cell death programs, including apoptosis, autophagy, pyroptosis, and ferroptosis. By controlling the expression of death-related genes and stress-response pathways, ncRNAs can determine whether tumor cells adapt to or succumb to therapeutic stress. In breast cancer, microRNAs (miRNAs), long non-coding RNAs (lncRNAs), and circular RNAs (circRNAs) have been shown to significantly influence tumor initiation, progression, metastasis, and therapeutic resistance [12]. Despite this mechanistic understanding, the clinical translation of ncRNAs remains limited. Currently, no reliable ncRNAs-based biomarkers exist for predicting DOX resistance risk, and no ncRNAs-mediated ferroptosis modulation strategies have advanced to clinical trials. This disparity underscores a significant gap between fundamental research and clinical application in this promising field.

Emerging evidence has revealed extensive functional crosstalk between ncRNAs and ferroptosis regulation. Multiple ncRNAs act as upstream modulators of key ferroptosis components, directly determining breast cancer cell susceptibility to this form of cell death through precise regulation of iron metabolism, lipid peroxidation, and antioxidant defense pathways [13,14]. These findings suggest that targeting specific ncRNAs could reactivate ferroptosis in tumor cells—effectively dismantling the defensive mechanisms underlying DOX resistance. The newly identified ncRNA-ferroptosis axis thus offers both a novel conceptual framework and promising therapeutic opportunities for fundamentally overcoming DOX resistance in breast cancer.

This review systematically examines the mechanisms by which ncRNAs overcome DOX resistance in breast cancer through regulation of core ferroptosis pathways. We begin by revisiting fundamental concepts of ncRNA biology, DOX resistance, and ferroptosis. The article subsequently details specific ncRNA-mediated regulatory networks that modulate ferroptosis to restore chemosensitivity, supported by updated experimental evidence. We then summarize recent advances in technological platforms and biomarker development in this field. Finally, through critical assessment of current research and existing controversies, this review proposes future translational directions. We anticipate that this comprehensive analysis will contribute to the understanding of chemoresistance mechanisms, facilitate the development of novel therapeutic strategies, and ultimately inform improved clinical management of breast cancer patients.

## 2 The non-coding RNAs-Ferroptosis axis in breast cancer: Foundational concepts and interplay with doxorubicin resistance

### 2.1 Classification and cancer-related functional roles of non-coding RNAs

Non-coding RNAs constitute a diverse class of RNA molecules that, despite lacking protein-coding capacity, play essential regulatory roles in numerous biological processes. Based on structural and functional characteristics, ncRNAs are primarily categorized into microRNAs (miRNAs), long non-coding RNAs (lncRNAs), and circular RNAs (circRNAs), among other subtypes [15]. miRNAs are small (∼22 nucleotides) RNA molecules that regulate gene expression at the post-transcriptional level through partial complementarity to the 3′-untranslated region (3′-UTR) of target mRNAs, resulting in translational repression or mRNA degradation [16]. LncRNAs, typically exceeding 200 nucleotides in length, exhibit more complex regulatory mechanisms involving epigenetic, transcriptional, and post-transcriptional modulation [17]. CircRNAs feature a covalently closed continuous loop structure that confers substantial stability against exonuclease degradation. These molecules function through diverse mechanisms including serving as competitive endogenous RNAs (ceRNAs) that sequester miRNAs, participating in transcriptional regulation, and modulating protein interactions [18]. While the exploration of ncRNAs in breast cancer remains relatively nascent, their established roles in other malignancies underscore their considerable potential for advancing our understanding of breast cancer biology.

Different classes of ncRNAs contribute distinctly to breast cancer pathogenesis and progression. Certain ncRNAs demonstrate oncogenic properties; for instance, lncRNA LINC00571 is significantly upregulated in breast cancer tissues where it promotes tumorigenesis by reprogramming tricarboxylic acid cycle metabolites, thereby enhancing proliferation, migration, and invasion in triple-negative breast cancer [19]. In contrast, other ncRNAs function as tumor suppressors. The miR-200 family, for example, effectively inhibits epithelial-mesenchymal transition (EMT), consequently impeding the metastatic capacity of breast cancer cells [20]. Thus, a systematic understanding of ncRNA classification and their molecular regulatory mechanisms not only advances comprehension of breast cancer pathogenesis but also identifies potential diagnostic biomarkers and therapeutic targets for clinical development.

From a functional standpoint, ncRNAs in breast cancer can be broadly classified as oncogenic or tumor-suppressive regulators, although their roles are often highly context-dependent. Oncogenic ncRNAs generally drive malignant phenotypes by promoting proliferation, invasion, metabolic reprogramming, stemness, and pro-survival signaling, whereas tumor-suppressive ncRNAs inhibit these processes and may enhance therapeutic responsiveness. Notably, increasing evidence indicates that ncRNAs are also intimately involved in therapeutic resistance through the regulation of drug efflux, apoptosis evasion, redox adaptation, DNA damage repair, and stress-response pathways. Rather than functioning in isolation, ncRNAs frequently operate within integrated regulatory networks, including ceRNA circuits and epigenetic regulatory axes, that collectively influence breast cancer progression and chemoresistance. This cancer-centered functional framework therefore provides a more appropriate basis for understanding how specific ncRNAs subsequently contribute to ferroptosis regulation and DOX resistance.

### 2.2 Molecular mechanisms of doxorubicin resistance in breast cancer

Doxorubicin remains a cornerstone chemotherapeutic agent in breast cancer treatment, though its clinical efficacy is frequently compromised by the development of drug resistance. As an anthracycline, DOX exerts cytotoxic effects through multiple interconnected mechanisms, including DNA intercalation, inhibition of topoisomerase II, generation of reactive oxygen species, and induction of replication-associated DNA damage. These events collectively disrupt genomic integrity and trigger tumor cell death, but they also create strong selective pressure for the emergence of resistant cell populations. The molecular mechanisms underlying DOX resistance in breast cancer are multifaceted, involving numerous signaling pathways and molecular determinants [21]. A primary resistance mechanism involves enhanced drug efflux mediated by ATP-binding cassette transporters, particularly P-glycoprotein (P-gp), which reduces intracellular DOX accumulation through active extrusion [6,22]. Recent investigations demonstrate that composite nanoparticles incorporating silver nanoparticles and DOX (Ag-TF@DOX) can counteract this mechanism by inhibiting P-gp ATPase activity, thereby restoring intracellular drug concentrations [23]. Furthermore, extracellular vesicles (EVs) derived from treatment-resistant breast cancer cells can horizontally transfer efflux transporters such as P-gp to drug-sensitive cells, propagating resistance within the tumor population [24].

Dysregulation of DNA damage repair represents another significant mechanism underlying DOX resistance. Studies indicate that methyltransferase 3 (METTL3) regulates DOX sensitivity by modulating homologous recombination (HR) repair efficiency. Genetic ablation of METTL3 in MCF-7 and MDA-MB-231 breast cancer cells resulted in substantial accumulation of DOX-induced DNA damage and impaired HR repair capacity, consequently enhancing DOX cytotoxicity [25]. Dysregulated DNA damage repair is particularly important in DOX resistance, as the cytotoxic effect of DOX largely depends on the persistence of DNA double-strand breaks and replication-associated stress. Breast cancer cells with enhanced homologous recombination capacity can repair DOX-induced genomic damage more efficiently, thereby attenuating apoptotic and growth-suppressive responses. In this context, the METTL3/EGF/RAD51 axis exemplifies how post-transcriptional regulation may enhance homologous recombination efficiency and reduce DOX sensitivity. More broadly, aberrant activation of DNA repair pathways enables resistant cells to withstand genotoxic stress and sustain survival during prolonged chemotherapy exposure. Additionally, disruption of intracellular redox homeostasis contributes critically to resistance development. GATA3 has been shown to promote DOX resistance by suppressing CYB5R2-mediated iron metabolism and interfering with ferroptosis execution, underscoring the pivotal role of iron metabolism in redox regulation [26]. In triple-negative breast cancer (TNBC), activation of antioxidant defense systems has been further demonstrated to facilitate metastasis-associated DOX resistance [27]. Elucidation of these molecular pathways provides novel theoretical foundations and potential therapeutic targets for overcoming DOX resistance and improving chemotherapeutic outcomes in breast cancer.

### 2.3 The role of ferroptosis in breast cancer

**Fig. 1:**
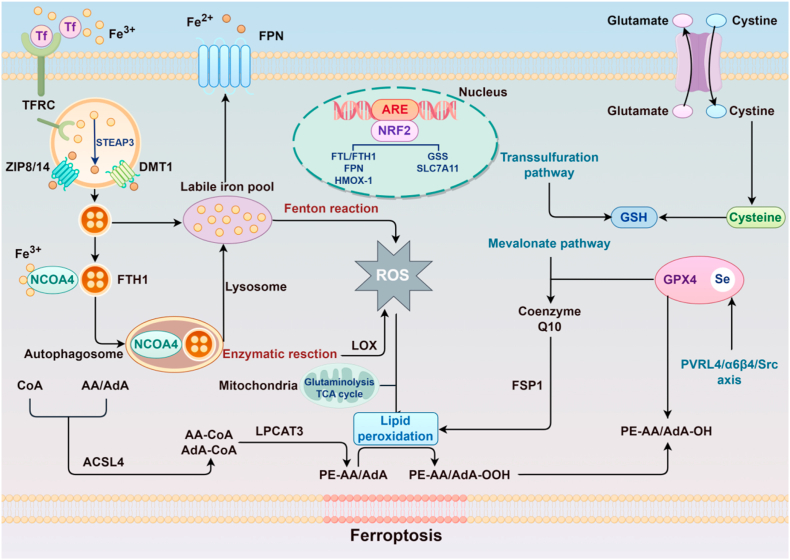
Molecular mechanisms of ferroptosis. Ferric iron (Fe^3+^) bound to transferrin (Tf) is imported into the cell via the transferrin receptor (TFRC) and reduced to ferrous iron (Fe^2+^) by STEAP3, thereby contributing to the labile iron pool. This pool fuels the generation of reactive oxygen species (ROS) via the Fenton reaction, a process further amplified by lysosomes, mitochondria, and lipoxygenases (LOXs). Mitochondria additionally enhance lipid peroxidation through glutaminolysis and the tricarboxylic acid (TCA) cycle. Intracellularly, cystine uptake is mediated by the cystine/glutamate antiporter system; it is subsequently reduced to cysteine and utilized for glutathione (GSH) synthesis. GSH acts as an essential cofactor for glutathione peroxidase 4 (GPX4), a selenoenzyme that reduces lipid peroxides. Moreover, the mevalonate pathway generates coenzyme Q10 (CoQ10), which cooperates with FSP1 to inhibit lipid peroxidation. Nuclear factor erythroid 2-related factor 2 (NRF2) binds to antioxidant response elements (ARE) and upregulates genes involved in iron metabolism, antioxidant defense, and cystine import (e.g., FTH1, SLC7A11). Other pathways, including the PVRL4/α6β4/Src signaling axis, also contribute to the regulatory network. The accumulation of lipid peroxidation products, such as PE-AA/Ada-OOH, ultimately triggers ferroptotic cell death.

Ferroptosis, an iron-dependent form of regulated cell death, plays a crucial role in breast cancer pathogenesis. This distinct cell death process is primarily mediated through three interconnected mechanisms: dysregulation of intracellular iron homeostasis, enhanced lipid peroxidation, and compromised antioxidant defense systems (Fig. 1). In breast cancer cells, aberrantly accumulated iron ions catalyze extensive lipid peroxidation via the Fenton reaction, generating excessive lipid peroxides. When this oxidative damage surpasses cellular antioxidant capacity, ferroptosis is initiated [28]. Experimental evidence demonstrates that combined administration of the ferroptosis inducers erastin and lapatinib significantly elevates intracellular iron levels in breast cancer cells, promoting substantial reactive oxygen species (ROS) accumulation and subsequent cell death. Importantly, this cytotoxic effect is substantially attenuated by the iron chelator deferoxamine [29], collectively validating the central role of ferroptosis in breast cancer cell death pathways.

Within the ferroptosis regulatory network of breast cancer, multiple key molecules orchestrate this cell death process through distinct yet interconnected pathways. The cystine/glutamate antiporter (system XC^−^) constitutes a crucial regulatory node, governing cellular susceptibility to ferroptosis by facilitating cystine uptake for glutathione (GSH) biosynthesis, thereby maintaining antioxidant capacity [30,31]. Experimental evidence demonstrates that Erastin, a specific system XC^−^ inhibitor, induces characteristic ferroptosis manifestations in MDA-MB-231 cells—including lipid peroxidation accumulation, iron overload, and mitochondrial condensation—through GSH depletion [32]. Iron metabolic homeostasis represents another central regulatory axis. Ferritin heavy chain 1 (FTH1), a key iron storage protein, negatively regulates ferroptosis sensitivity, as evidenced by FTH1 overexpression suppressing BY1-induced ferroptosis while its knockdown enhances cellular vulnerability. Mechanistically, BY1 promotes NCOA4-FTH1 interaction, increasing intracellular ferrous iron availability and driving ferroptosis execution [33]. Notably, ferroptosis regulation exhibits molecular subtype specificity in breast cancer. In estrogen receptor-positive (ER+) subtypes, ERα functions as a ferroptosis suppressor—its ablation induces ferroptosis, reversible by the inhibitor Fer-1(34). Mechanistically, ERα-mediated signaling may protect ER-positive breast cancer cells from ferroptotic death by sustaining antioxidant capacity and limiting the accumulation of toxic lipid peroxides, thereby contributing to subtype-specific ferroptosis resistance. Heat shock protein B1 (HSPB1) confers resistance by attenuating lipid peroxidation, with its upregulation associated with chemoresistance; HSPB1 inhibition consequently enhances ferroptosis inducer efficacy [34]. Furthermore, ferroptosis suppressor protein 1 (FSP1) mediates protection through the FSP1-coenzyme Q10-NAD(P)H axis and vitamin K redox cycling, regulated transcriptionally and epigenetically including through ncRNAs [35]. These findings collectively delineate a sophisticated ferroptosis regulatory network in breast cancer, advancing our understanding of tumor cell death mechanisms while providing a conceptual framework for developing novel therapeutic strategies centered on ferroptosis induction. These observations indicate that ferroptosis regulation is closely linked to chemotherapy adaptation, and that suppression of ferroptotic vulnerability may represent an important mechanism underlying DOX resistance in breast cancer.

### 2.4 Common molecular pathways of ncRNA-Ferroptosis axis in regulating doxorubicin resistance in breast cancer

**Fig. 2:**
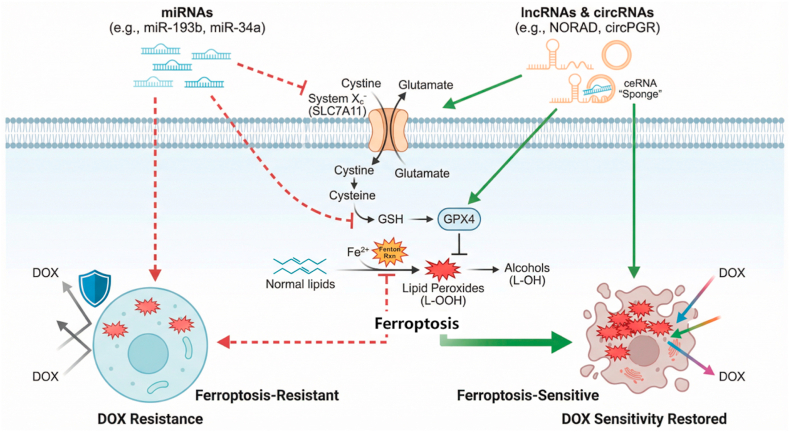
Schematic representation of the ncRNAs–ferroptosis regulatory axis in DOX-resistant breast cancer. This diagram depicts the multi-tiered regulatory network through which ncRNAs modulate ferroptosis to influence doxorubicin sensitivity in breast cancer cells. Specifically, microRNAs (miRNAs), including miR-193b and miR-34a, directly bind to the 3′-untranslated regions of ferroptosis-related mRNAs such as MCL-1 and SLC7A11, thereby repressing their expression post-transcriptionally and subsequently promoting ferroptosis and enhancing DOX chemosensitivity. Conversely, long non-coding RNAs (lncRNAs) like NORAD and circular RNAs (circRNAs) such as circPGR primarily act as competing endogenous RNAs (ceRNAs) or molecular sponges that sequester miRNAs, leading to the upregulation of core ferroptosis regulators including SLC7A11 and GPX4. This cascade reinforces the System Xc^−^–GSH–GPX4 antioxidant axis, suppresses lipid peroxide accumulation, and fosters a ferroptosis-resistant phenotype, ultimately contributing to DOX resistance. The dynamic equilibrium between these opposing regulatory mechanisms dictates the cellular susceptibility to ferroptosis, thereby determining the therapeutic efficacy of DOX.

Despite the diversity of ncRNA species, their regulatory effects on ferroptosis-associated DOX resistance in breast cancer converge on several common molecular pathways, including antioxidant defense, iron metabolism, lipid peroxidation, and redox-adaptive signaling. Different classes of ncRNAs—including miRNAs, lncRNAs, and circRNAs—collectively modulate DOX resistance in breast cancer through coordinated regulation of core ferroptosis pathways, forming a multi-layered molecular network (Fig. 2). While sharing the common objective of regulating ferroptosis sensitivity, these ncRNA categories employ distinct yet complementary mechanisms, establishing an integrated regulatory system.

microRNAs (miRNAs) mediate rapid post-transcriptional control through direct targeting of key ferroptosis-related mRNAs. For example, miR-193b binds the 3′-UTR of MCL-1 mRNA, inhibiting this anti-apoptotic protein's expression and consequently relieving ferroptosis suppression to enhance DOX sensitivity [36]. Similarly, miR-34a directly targets SLC7A11, a core component of System Xc^−^, thereby reducing cystine uptake and glutathione synthesis while promoting lipid peroxide accumulation—effectively reversing DOX resistance [14]. Such miRNAs are frequently downregulated in DOX-resistant cells, and their restoration significantly improves chemotherapeutic efficacy, designating them as potential "anti-resistance" agents.

In contrast, long non-coding RNAs (lncRNAs) modulate ferroptosis through more complex mechanisms, primarily via competitive endogenous RNA (ceRNA) networks and epigenetic regulation. Within ceRNA networks, lncRNAs act as molecular sponges that sequester specific miRNAs, thereby attenuating their repression of target mRNAs. For instance, lncRNA NORAD enhances System Xc^−^ function by relieving its inhibition of SLC7A11, thereby suppressing ferroptosis and promoting DOX resistance [37]. Similarly, lncRNA MAFG-AS1 stabilizes the XCT/GSH/GPX4 antioxidant axis, counteracting the effects of ferroptosis inducers; its elevated expression correlates positively with DOX resistance [38]. At the epigenetic level, certain lncRNAs recruit chromatin-modifying complexes to regulate gene expression. LncRNA STMN1P2, for example, interacts with hnRNPU protein to enhance EZH2 stability, leading to transcriptional repression of ferroptosis-related genes and ultimately facilitating drug resistance development [39].

circRNAs, characterized by their covalently closed circular structure and exceptional stability, function as sustained regulators in the ferroptosis network. While primarily operating through competitive endogenous RNA (ceRNA) mechanisms, their inherent stability enables prolonged regulatory effects. For instance, circPGR promotes DOX resistance in estrogen receptor-positive breast cancer by sequestering miRNAs to upregulate GPX4 expression, thereby enhancing cellular antioxidant capacity [40]. Additionally, studies demonstrate that a specific circRNA acts as a molecular sponge for miR-185-5p, relieving its suppression of the key iron transporter gene SLC25A28. The subsequent upregulation of SLC25A28 increases mitochondrial iron import, ensuring iron availability for heme and iron-sulfur cluster biosynthesis while disturbing cellular iron homeostasis. This imbalance indirectly modulates the ferroptosis pathway, ultimately contributing to tumor drug resistance [41].

In summary, distinct classes of ncRNAs collectively orchestrate ferroptosis regulation through complementary mechanisms: miRNAs enable rapid target-specific control, lncRNAs facilitate multi-level regulatory integration, and circRNAs provide sustained modulation through their structural stability. Dysregulation of this coordinated network represents a crucial determinant of DOX resistance, while its targeted restoration offers a promising strategic approach for resensitizing resistant tumours. Elucidation of this sophisticated regulatory framework provides a critical foundation for developing novel ncRNA-directed therapeutic interventions against chemoresistant breast cancer. For clarity, the major representative miRNAs, lncRNAs, and circRNAs implicated in these pathways are comparatively summarized in Table 1.

**Table 1:** Representative ncRNAs involved in ferroptosis-associated doxorubicin resistance in breast cancer.

Type	ncRNA	Major target/pathway	Effect on ferroptosis and DOX response	Reference
miRNA	miR-193b	MCL-1	Promotes ferroptosis and increases DOX sensitivity by suppressing MCL-1	37
	miR-34a	SLC7A11/System Xc^-^	Inhibits SLC7A11, reduces GSH production, promotes lipid peroxidation, and reverses DOX resistance	14
lncRNA	NORAD	FUS/NR3C1/SLC7A11 axis	Suppresses ferroptosis and promotes DOX resistance by upregulating SLC7A11	38
	MAFG-AS1	XCT/GSH/GPX4 antioxidant axis	Stabilizes antioxidant defenses, inhibits ferroptosis, and promotes DOX resistance	39
	STMN1P2	EZH2-related regulatory pathway	Contributes to chemoresistance; silencing restores DOX sensitivity and may facilitate ferroptosis-related cell death	40
	PVT1	Keap1-Nrf2 pathway	Enhances antioxidant capacity, suppresses ferroptosis, and reduces DOX cytotoxicity	54
	LncFASA	Multiple ferroptosis-related pathways	Increases ferroptosis susceptibility and may help overcome DOX resistance in TNBC	56
circRNA	circPGR	GPX4	Upregulates GPX4 via ceRNA activity, suppresses ferroptosis, and promotes DOX resistance	41

## 3 Clinical translation and current research status of non-coding RNA targeting ferroptosis to reverse DOX resistance

### 3.1 The value of non-coding RNAs as biomarkers in breast cancer

Non-coding RNAs have emerged as critical tools for the diagnosis, molecular subtyping, and prognostic evaluation of breast cancer, largely due to their tissue specificity and high stability in body fluids. At the tissue level, numerous studies have established close associations between specific ncRNAs and breast cancer pathogenesis. For instance, the lncRNA BC069792 has been shown to possess tumor-suppressive functions, with its aberrant expression in breast cancer tissues closely linked to tumor progression; both in vitro and in vivo studies support its potential utility as a diagnostic biomarker [42]. Similarly, lncRNA FGF14-AS2 is significantly downregulated in breast cancer, and its overexpression effectively inhibits cell proliferation and migration while inducing apoptosis, suggesting its role as both a tumor suppressor and a prognostic indicator [43]. In addition, genetic polymorphisms in lncRNA MEG3 have been significantly correlated with response to neoadjuvant chemotherapy and long-term survival, positioning it as a promising molecular marker for individualized efficacy prediction and prognosis [44].

In the context of liquid biopsy, circulating ncRNAs offer distinct advantages for non-invasive diagnosis. Plasma microRNA-195, microRNA-34c, and microRNA-1246 have been identified as novel biomarkers for diagnosing HER2-positive breast cancer with resistance to trastuzumab [45]. Moreover, the estrogen-induced circular RNA, circPGR, plays a key regulatory role in the growth of ER-positive breast cancer cells; its expression level not only reflects the activity of estrogen signaling pathways but also serves as an effective predictive marker of therapeutic response [40]. LncRNA 01087 is differentially expressed between luminal-type and triple-negative breast cancer, and its expression level significantly correlates with patient survival outcomes, underscoring its potential as a prognostic biomarker [46].

### 3.2 Preclinical research on non-coding RNAs as therapeutic targets

In the realm of therapeutic target development, ncRNAs present novel avenues for overcoming drug resistance and enhancing treatment efficacy in breast cancer. A growing body of evidence indicates that various ncRNAs play central roles in regulating malignant phenotypes—including proliferation, metastasis, and chemoresistance—through the modulation of key signaling pathways [47,48]. Notably, miR-193b is significantly upregulated in DOX-sensitive breast cancer cells and enhances tumor cell sensitivity to DOX by targeting and inhibiting the anti-apoptotic gene MCL-1, thereby promoting ferroptosis [36]. This finding provides a theoretical basis for sensitization strategies employing miRNA mimics.

Targeted inhibition of oncogenic ncRNAs also demonstrates considerable therapeutic potential. LncRNA TINCR facilitates breast cancer progression by mediating tumor immune evasion; research indicates that its targeted inhibition can substantially enhance the anti-tumor efficacy of PD-L1 inhibitors, offering a novel strategy to reverse immunotherapy resistance [49]. In triple-negative breast cancer, the highly expressed lncRNA CDKN2B-AS1 exerts oncogenic effects by regulating cyclin-dependent kinase inhibitors such as p15INK4b, and molecular intervention targeting this axis may represent a specific therapeutic approach to overcome DOX resistance [50]. Additionally, microRNAs implicated in inflammatory breast cancer have been identified as potential oncogenic or tumor-suppressive targets [51].

Adding to this complexity, ncRNAs contribute to the establishment of chemoresistance through the formation of intricate regulatory networks. An lncRNA/miRNA/mRNA regulatory axis identified in MCF7 drug-resistant cell lines has been confirmed to be closely associated with tamoxifen resistance. Targeted intervention against this pathway offers a promising therapeutic direction for reversing chemoresistance in estrogen receptor-positive breast cancer [52].

### 3.3 Research on non-coding RNAs in reversing doxorubicin resistance

Preclinical studies have increasingly elucidated the pivotal role of specific ncRNAs in regulating DOX resistance in breast cancer, with their underlying molecular mechanisms offering novel targets for reversing chemoresistance. For instance, lncRNA STMN1P2 is significantly overexpressed in doxorubicin-resistant breast cancer tissues and cell models. Functional experiments have confirmed that knockdown of STMN1P2 effectively reduces resistance to doxorubicin in breast cancer cells. Mechanistically, STMN1P2 interacts with the hnRNPU protein to enhance the protein stability of EZH2, which subsequently inhibits the activation of tumor necrosis factor receptor-associated factor 6 (TRAF6). This suppression ultimately blocks DOX-induced pyroptosis, thereby promoting a chemoresistant phenotype. In an MCF7/DOX cell xenograft tumor model, targeted silencing of STMN1P2 significantly enhanced the inhibitory effect of DOX on tumor growth [39]. Similarly, lncRNA PVT1 has been implicated in DOX resistance in triple-negative breast cancer. In MDA-MB-231 cells, PVT1 interferes with the binding between kelch-like ECH-associated protein 1 (Keap1) and nuclear factor erythroid 2-related factor 2 (Nrf2), thereby enhancing Nrf2 protein stability and activating its downstream anti-oxidative stress pathway. This attenuates the cytotoxic effects of DOX and contributes to the development of drug resistance [53]. Collectively, these studies not only reveal the specific pathways through which ncRNAs regulate DOX resistance—ranging from epigenetic regulation to oxidative stress response—but also validate the feasibility of targeting ncRNAs to reverse chemotherapy resistance. These findings provide critical experimental evidence and translational insights for the development of combination therapeutic strategies aimed at improving prognosis in patients with breast cancer.

### 3.4 Current research status of non-coding RNAs in regulating ferroptosis

The role of ncRNAs in regulating ferroptosis has emerged as a focal point in tumor biology, with substantial progress achieved across various malignancies, including breast cancer. Accumulating evidence indicates that specific ncRNAs critically influence ferroptosis through the fine-tuned modulation of iron metabolism and redox homeostasis. In breast cancer, for example, lncRNA MEG8 functions as a molecular sponge for miR-497-5p, thereby alleviating its inhibitory effect on the target gene NOTCH2. This regulatory axis modulates downstream ferroptosis-related pathways, ultimately suppressing tumor cell proliferation and promoting ferroptosis [54]. Recent studies have demonstrated that the long non-coding RNA LncFASA significantly enhances the susceptibility of triple-negative breast cancer (TNBC) to ferroptosis through the concurrent regulation of multiple core ferroptosis pathways [55]. This finding suggests that LncFASA may serve not only as a potential biomarker for predicting the response of TNBC patients to ferroptosis-inducing therapies but also offers a novel strategy for reversing treatment resistance by modulating lncRNA expression levels. In other tumor types, such as head and neck squamous cell carcinoma, prognostic models constructed based on the expression profiles of ferroptosis-related lncRNAs have demonstrated robust clinical stratification capability, further underscoring the systemic involvement of ncRNAs within ferroptosis regulatory networks [56].

Despite these advances, the precise molecular networks through which ncRNAs govern ferroptosis remain to be comprehensively delineated. Current investigations predominantly focus on interactions between individual ncRNAs and a limited subset of ferroptosis-associated genes, with a unified regulatory framework yet to be established [57,58]. Moreover, translating these fundamental discoveries into clinically viable targeted therapies continues to face significant technical hurdles, including delivery system efficiency, tissue specificity, and in vivo stability. Future research should integrate multi-omics approaches with gene editing technologies to elucidate the global regulatory mechanisms of ncRNAs in ferroptosis. Concurrently, efforts must be directed toward developing efficient and safe targeted delivery systems, thereby advancing ncRNA-based ferroptosis modulation from theoretical constructs toward clinical application and offering transformative strategies for cancer therapy.

## 4 Technological strategies supporting the investigation and translation of the ncRNA-ferroptosis axis in doxorubicin-resistant breast cancer

Emerging technological platforms are accelerating both mechanistic investigation and translational advancement of the ncRNA-ferroptosis axis in DOX-resistant breast cancer. High-throughput sequencing provides a robust foundation for identifying differentially expressed miRNAs and lncRNAs during the development of resistance, thereby nominating candidate molecules for subsequent functional evaluation [59,60]. Bioinformatic analyses further facilitate this process by reconstructing ncRNA–target regulatory networks and prioritizing key pathways for experimental validation; for example, computational prediction identified MCL-1 as a target of miR-193b, and functional assays confirmed that miR-193b regulates DOX sensitivity through modulation of this anti-apoptotic factor [36,61]. In parallel, CRISPR-based approaches enable direct functional interrogation of ncRNAs and systematic screening of resistance-associated regulators, thereby accelerating the identification of actionable nodes within the ferroptosis network [[62], [63], [64]].

Beyond target discovery, post-transcriptional intervention and delivery technologies are critical for therapeutic translation. RNA interference remains a practical strategy for silencing oncogenic ncRNAs and assessing their contribution to ferroptosis sensitivity. For instance, RNAi-mediated inhibition of lncRNA NORAD suppresses SLC7A11 through the FUS/NR3C1 pathway and enhances erastin-induced ferroptosis in breast cancer cells [37], while related studies in other tumor models further support the feasibility of RNA-based ferroptosis modulation [41]. In addition, nanotechnology-based delivery systems, including lipid nanoparticles and stimulus-responsive liposomes, improve the precision and efficacy of ncRNA-targeted interventions by enhancing tumor-selective accumulation and reducing off-target exposure [65,66]. Collectively, these platforms constitute an integrated methodological pipeline spanning target discovery, functional validation, and therapeutic delivery, thereby supporting the translational development of ncRNA-mediated ferroptosis targeting as a strategy to reverse DOX resistance in breast cancer.

## 5 Controversies and challenges of non-coding RNA-targeted ferroptosis in doxorubicin-resistant breast cancer

### 5.1 Challenges posed by mechanistic complexity: Redundancy and heterogeneity of the regulatory network

While targeting non-coding RNAs to modulate ferroptosis represents a promising strategy for overcoming chemoresistance in breast cancer, both the complexity of the ferroptosis regulatory network and the intrinsic heterogeneity of tumours pose substantial hurdles to its clinical translation [67,68]. A key challenge is the high degree of redundancy and compensatory signalling embedded within this network, which inherently limits the effectiveness of single-node interventions. Ferroptosis execution requires the concerted dysregulation of iron homeostasis, accumulation of lipid peroxides, and collapse of antioxidant defences. However, tumour cells have evolved multilayered protective mechanisms. For example, when the classical glutathione peroxidase 4 (GPX4)-dependent antioxidant pathway is inhibited, cells can rapidly engage compensatory systems such as ferroptosis suppressor protein 1 (FSP1), which regenerates coenzyme Q10 to neutralise lipid peroxyl radicals and preserve redox balance [35,69]. This synergistic and compensatory interplay between GPX4 and FSP1 implies that simply modulating a single ncRNA to up- or down-regulate one arm of the pathway (e.g., circPGR-mediated upregulation of GPX4) may not suffice to trigger robust ferroptosis; instead, such an approach could inadvertently select for more resistant cell subpopulations [40]. Compounding this issue, sensitivity to ferroptosis differs markedly across breast cancer molecular subtypes, underscoring the need for tailored ncRNA-based interventions. Triple-negative breast cancer typically exhibits high metabolic activity, elevated reactive oxygen species, and active iron turnover, rendering it relatively susceptible to ferroptosis inducers [70,71]. By contrast, hormone receptor-positive (Luminal) tumours often upregulate the system Xc^−^ subunit SLC7A11 via oestrogen receptor α signalling, thereby enhancing cystine uptake and glutathione synthesis and building a robust antioxidant shield [72]. Consequently, inducing ferroptosis in Luminal breast cancer by targeting ncRNAs—for instance, using miR-34a to suppress SLC7A11—may require concomitant application of stronger oxidative stressors or simultaneous blockade of alternative salvage pathways such as FSP1(71). Moreover, ncRNA regulation itself is characterised by substantial cellular heterogeneity and dynamic variability [73]. The expression level and functional impact of a given ncRNA can vary widely among breast cancer cells with different genetic backgrounds or exposed to distinct microenvironmental cues. Although lncRNA NORAD has been repeatedly shown to inhibit ferroptosis and foster drug resistance, the binding efficiency and downstream consequences of its intricate competing endogenous RNA (ceRNA) network remain inconsistent across cell lines [37]. In parallel, microenvironmental factors—including hypoxia, acidic pH, and inflammatory cytokines—can dynamically tune ncRNA expression, thereby modulating the transient susceptibility of tumour cells to ferroptotic stimuli [74]. This spatiotemporal heterogeneity suggests that investigations relying on a single model system or a solitary time point are unlikely to capture the full spectrum of ncRNA function in vivo, highlighting the necessity for more physiologically relevant and temporally resolved experimental approaches.

### 5.2 Therapeutic safety and translational hurdles: Barriers from the molecular to the systemic level

Beyond the inherent mechanistic complexity, translating ncRNA-based ferroptosis modulation into clinical practice for reversing DOX resistance must also surmount a series of safety and technical bottlenecks that span molecular design, formulation, and systemic administration [75]. A principal obstacle is the off-target effects and immunogenicity intrinsic to ncRNA therapeutics [76]. Most therapeutic ncRNAs operate via RNA interference; however, partial sequence homology can lead to unintended binding to the 3′-untranslated regions of non-target mRNAs, resulting in off-target gene silencing [77]. Such off-target events may disrupt normal cellular functions and, in some cases, even harbour pro-tumorigenic risks. Moreover, synthetic ncRNAs—particularly those bearing chemical modifications—are susceptible to recognition by pattern recognition receptors, which can instigate innate immune responses. Clinical trials with certain siRNA candidates have already demonstrated dose-limiting toxicities driven by robust interferon induction and inflammatory reactions. Hence, a persistent challenge in drug design is to balance stability with immunogenicity through precise chemical modifications (e.g., 2′-O-methyl substitutions or phosphorothioate linkages) that minimise immune recognition without compromising activity [78]. A second major hurdle concerns the suboptimal efficacy and safety of current targeted delivery systems [79]. ncRNA molecules are negatively charged, relatively large, and prone to nuclease degradation, necessitating formulation within carriers such as lipid nanoparticles, polymeric micelles, or exosomes for in vivo application. Nevertheless, most existing platforms suffer from two interrelated drawbacks [65]. First, pronounced hepatic and splenic uptake by the mononuclear phagocyte system leads to off-target accumulation, reducing the bioavailability at tumour sites and raising concerns about hepatotoxicity [76]. Second, the long-term toxicity of the carriers themselves remains inadequately characterised. While cationic lipids and polymers facilitate efficient encapsulation and endosomal escape, their positive charge can compromise plasma membrane integrity, induce mitochondrial damage, or trigger autophagy; moreover, the metabolic fate and potential cumulative toxicity of their degradation products in vivo are still poorly understood [75,80]. A third, equally formidable challenge stems from tumour evolution and adaptive resistance, which threaten the durability of therapeutic responses [81]. Even when initial treatment proves effective, the selective pressure exerted by ncRNA agents can drive the emergence of resistant clones through diverse mechanisms. For instance, following administration of miRNA mimics intended to restore tumour-suppressive function, cancer cells may silence the corresponding endogenous miRNA via promoter methylation or upregulate downstream targets to circumvent inhibition [82]. In addition, non-malignant cells within the tumour microenvironment, such as cancer-associated fibroblasts, can transfer resistance-conferring ncRNAs to malignant cells via exosomes, thereby propagating a resistant phenotype throughout the tumour mass [24,83]. This co-evolution within the tumour ecosystem poses a substantial challenge to interventions focused on a single molecular node.

In summary, overcoming the clinical bottlenecks associated with ncRNA-regulated ferroptosis in the context of DOX resistance demands a conceptual shift from static, single-mechanism descriptions toward dynamic systems-level analyses. Future efforts should integrate high-throughput screening, single-cell genomics, and advanced in vivo imaging to precisely delineate how different breast cancer subtypes adapt under therapeutic pressure. Building on this systems-level understanding, the next generation of smart nanomedicines—endowed with improved safety profiles, more accurate tumour targeting, and the capacity for synergistic multi-node intervention—can be rationally designed and evaluated [[84], [85], [86]].

## 6 Future perspectives on ncRNAs, ferroptosis, and doxorubicin resistance in breast cancer

### 6.1 Deconstructing complexity: From single-target regulation to network intervention

Given the extensive redundancy and compensatory circuitry inherent to the ferroptosis regulatory network, future investigations must evolve beyond linear descriptions of individual non-coding RNAs toward a systematic dissection of their integrated regulatory architecture across distinct breast cancer molecular subtypes [67,68]. Interventions aimed at a single nodal point are particularly vulnerable to failure because tumour cells can rapidly engage alternative pathways—exemplified by the swift upregulation of FSP1-mediated antioxidant defences upon GPX4 inhibition [69]. A pivotal research direction, therefore, is the construction of breast cancer-specific ncRNA–ferroptosis regulatory maps through the integration of high-throughput screening, multi-omics profiling, and functional genomics [73,87]. Such maps would enable the identification of “critical nodes” that are functionally relevant in specific subtypes or drug-resistant stages. Building on this systems-level knowledge, the rational design of combination strategies that simultaneously engage multiple core nodes—for example, co-targeting ncRNAs implicated in both GPX4 and FSP1 pathways, or pairing ncRNA mimics with direct ferroptosis inducers—holds considerable promise for synergistically dismantling the antioxidant machinery of tumour cells. These multi-pronged approaches may effectively circumvent network buffering and compensatory resilience, thereby achieving more robust and durable ferroptosis induction [57,88].

An important direction for future research is to move beyond the descriptive cataloging of ncRNA-mediated regulators of ferroptosis and toward a systems-level understanding of how these molecules determine ferroptotic susceptibility in DOX-resistant breast cancer. Within this framework, the ncRNA–ferroptosis axis may be more appropriately viewed as a dynamic adaptive network that coordinates iron metabolism, lipid peroxidation, antioxidant defense, and chemotherapy-induced stress signaling. This perspective suggests that effective reversal of DOX resistance may rely not on targeting a single ncRNA or ferroptosis-associated factor, but rather on identifying subtype-specific ferroptotic vulnerabilities and modulating critical regulatory nodes. Accordingly, future investigations should emphasize multi-omics integration, subtype-specific functional validation, biomarker discovery, and clinically applicable delivery platforms to facilitate the translation of ncRNA-mediated ferroptosis modulation into precision therapeutic approaches.

### 6.2 Overcoming translational hurdles: Developing intelligent and safe precision delivery systems

The successful clinical translation of ncRNA-based therapeutics is inextricably linked to fundamental advances in delivery technology. To surmount the persistent challenges of off-target effects, immunogenicity, and carrier-related toxicity, future efforts must concentrate on the development of intelligent nano-delivery systems endowed with superior targeting precision, biocompatibility, and spatiotemporal controllability [75]. A multi-pronged strategy can be envisaged: first, the functionalisation of nanoparticles with ligands that actively recognise and bind to receptors overexpressed on breast cancer cells, thereby enhancing tumour-specific uptake; second, the design of environmentally responsive carriers that leverage tumour microenvironmental cues (e.g., acidic pH, hypoxia, or specific enzymes) to trigger cargo release selectively at the tumour site, thus minimising hepatic entrapment and off-tissue distribution [66,85]; third, the refinement of chemical modification schemes—such as judicious incorporation of 2′-O-methyl groups or phosphorothioate linkages—that preserve ncRNA stability while effectively dampening innate immune recognition [78]; and fourth, the exploration of endogenous delivery vehicles, notably engineered exosomes, which may circumvent the long-term safety concerns associated with synthetic nanomaterials and offer a more physiologically compatible platform for ncRNA transport [78,79]. Collectively, these approaches aim to establish a robust foundation for the clinical safety and efficacy of ncRNA therapeutics.

### 6.3 Innovating therapeutic modalities: Constructing a new paradigm of multimodal synergistic therapy

Given the capacity of tumours to evolve and adapt under therapeutic pressure, interventions relying on a single ncRNA agent are unlikely to achieve durable efficacy [81]. Future progress therefore hinges on the strategic integration of ncRNA-based approaches with established treatment modalities to establish a new paradigm of multimodal synergistic therapy. On one front, ncRNA modulation can function as a sensitising strategy—for example, using miR-34a mimics to suppress SLC7A11, thereby attenuating the antioxidant capacity of tumour cells and rendering them more vulnerable to subsequent chemotherapy or ferroptosis inducers [14,89]. Concurrently, the induction of ferroptosis itself may remodel the tumour immune microenvironment; the release of damage-associated molecular patterns during ferroptotic cell death can facilitate dendritic cell maturation and enhance T cell infiltration [90]. This rationale supports combining ncRNA-targeted interventions with immune checkpoint inhibitors, as ferroptosis-mediated immunogenic cell death could help convert immunologically "cold" tumours into "hot" ones, thereby amplifying anti-tumour immune responses in a synergistic manner [49,91]. Moreover, the incorporation of gene editing technologies to directly rectify resistance-associated ncRNAs or their cognate target genes presents an additional avenue for fundamentally reversing drug resistance [62,64,92]. Through such multidimensional, multi-mechanistic combinatorial approaches, it may become feasible to surmount tumour adaptive evolution and deliver more sustained clinical benefits for breast cancer patients confronting DOX resistance [84,93].

## 7 Conclusion

In conclusion, ncRNAs play a critical role in regulating DOX resistance in breast cancer through multilayered modulation of ferroptosis-related pathways. By affecting key processes such as iron metabolism, lipid peroxidation, and antioxidant defense—particularly the System Xc^−^-GSH-GPX4 axis, the FSP1-CoQ10 pathway, and iron homeostasis—miRNAs, lncRNAs, and circRNAs collectively influence ferroptosis susceptibility and chemotherapeutic response (Table 1). Current evidence suggests that targeting this ncRNA–ferroptosis regulatory network may offer both mechanistic insight and translational potential for overcoming DOX resistance, with possible applications in biomarker development and therapeutic intervention. Nevertheless, substantial challenges remain for clinical translation, including regulatory network complexity, molecular subtype heterogeneity, delivery efficiency, and biosafety. Continued integration of multi-omics analyses, functional validation, and precise delivery strategies will be essential to advance ncRNA-based ferroptosis modulation toward meaningful clinical application in breast cancer.

## CRediT authorship contribution statement

**Xiaojie Yu:** Writing – review & editing, Writing – original draft, Validation, Supervision, Methodology, Data curation, Conceptualization. **An Xu:** Visualization, Conceptualization. **Longdi Yao:** Data curation, Conceptualization. **Xiao Huang:** Formal analysis. **Jianwen Wang:** Conceptualization. **Deyuan Fu:** Writing – original draft, Visualization, Funding acquisition.

## Funding

This work was supported by the 10.13039/501100001809National Natural Science Foundation of China (No.82072909).

## Declaration of competing interest

The authors declare that they have no known competing financial interests or personal relationships that could have appeared to influence the work reported in this paper.
